# Examining the Gateway Hypothesis and Mapping Substance Use Pathways on Social Media: Machine Learning Approach

**DOI:** 10.2196/54433

**Published:** 2024-05-07

**Authors:** Yunhao Yuan, Erin Kasson, Jordan Taylor, Patricia Cavazos-Rehg, Munmun De Choudhury, Talayeh Aledavood

**Affiliations:** 1 Department of Computer Science Aalto University Espoo Finland; 2 School of Medicine Washington University in St. Louis St. Louis, MO United States; 3 Carnegie Mellon University Pittsburgh, PA United States; 4 Georgia Institute of Technology Atlanta, GA United States

**Keywords:** gateway hypothesis, substance use, social media, deep learning, natural language processing

## Abstract

**Background:**

Substance misuse presents significant global public health challenges. Understanding transitions between substance types and the timing of shifts to polysubstance use is vital to developing effective prevention and recovery strategies. The gateway hypothesis suggests that high-risk substance use is preceded by lower-risk substance use. However, the source of this correlation is hotly contested. While some claim that low-risk substance use causes subsequent, riskier substance use, most people using low-risk substances also do not escalate to higher-risk substances. Social media data hold the potential to shed light on the factors contributing to substance use transitions.

**Objective:**

By leveraging social media data, our study aimed to gain a better understanding of substance use pathways. By identifying and analyzing the transitions of individuals between different risk levels of substance use, our goal was to find specific linguistic cues in individuals’ social media posts that could indicate escalating or de-escalating patterns in substance use.

**Methods:**

We conducted a large-scale analysis using data from Reddit, collected between 2015 and 2019, consisting of over 2.29 million posts and approximately 29.37 million comments by around 1.4 million users from subreddits. These data, derived from substance use subreddits, facilitated the creation of a risk transition data set reflecting the substance use behaviors of over 1.4 million users. We deployed deep learning and machine learning techniques to predict the escalation or de-escalation transitions in risk levels, based on initial transition phases documented in posts and comments. We conducted a linguistic analysis to analyze the language patterns associated with transitions in substance use, emphasizing the role of n-gram features in predicting future risk trajectories.

**Results:**

Our results showed promise in predicting the escalation or de-escalation transition in risk levels, based on the historical data of Reddit users created on initial transition phases among drug-related subreddits, with an accuracy of 78.48% and an *F*_1_-score of 79.20%. We highlighted the vital predictive features, such as specific substance names and tools indicative of future risk escalations. Our linguistic analysis showed that terms linked with harm reduction strategies were instrumental in signaling de-escalation, whereas descriptors of frequent substance use were characteristic of escalating transitions.

**Conclusions:**

This study sheds light on the complexities surrounding the gateway hypothesis of substance use through an examination of web-based behavior on Reddit. While certain findings validate the hypothesis, indicating a progression from lower-risk substances such as marijuana to higher-risk ones, a significant number of individuals did not show this transition. The research underscores the potential of using machine learning with social media analysis to predict substance use transitions. Our results point toward future directions for leveraging social media data in substance use research, underlining the importance of continued exploration before suggesting direct implications for interventions.

## Introduction

### Background

Substance misuse has become a major public health challenge around the globe, leading to a considerable health burden and substantial financial loss. For example, in the United States, drug-involved overdoses driven by fentanyl and other opioids resulted in the loss of nearly 106,000 lives in 2021 [1].

Understanding the dynamic transitions between types of substances used and the timing of transitions to polysubstance use is vital for developing effective prevention, harm reduction, and recovery strategies. Since the 1970s, researchers have explored the *gateway hypothesis*, a theory suggesting a structured, sequential pattern of drug involvement among individuals, often starting with the use of substances such as alcohol, tobacco, or cannabis and potentially progressing to the use of more potent substances with higher associated risks [2]. Despite the long-standing prevalence of the gateway hypothesis in the literature, recent studies [3,4] have begun to scrutinize its validity. While the gateway hypothesis may explain specific substance use patterns, it does not adequately capture the “causal” or “direct” relationships. Moreover, it is not able to describe complex and multidirectional patterns of substance use. Nevertheless, this does not diminish the significance of the gateway framework. At the macrolevel, patterns have been identified where higher-risk substance use is often preceded by lower-risk substance use [5]. While many individuals might begin their substance use journey with alcohol or marijuana, it does not imply an inevitable progression to substances like opiates. Most users of marijuana do not escalate to using opiates [6]. However, this raises important questions about what microlevel factors differentiate substance use escalation and de-escalation. More information is needed to understand these microlevel risk factors associated with substance use escalation and de-escalation. Identifiable risk factors exist among groups that engage in substance use, potentially increasing the likelihood of transitioning from one substance to another, and the gateway hypothesis framework could be evaluated and expanded upon given new sources of data (ie, social media). Given the complex nature of substance use initiation and people’s highly individualized journeys, understanding common trajectories of substances used is essential for informing substance use intervention programs and for preventing escalation to misuse behaviors and substances associated with higher risk (ie, overdose).

There has been extensive work on developmental stages in substance use involvement using survey data [7-9]. For instance, taking cannabis as a gateway drug case study, recent research from the National Institute on Drug Abuse in the United States confirms that marijuana use typically precedes *harder* drug use, but most marijuana users do not escalate to *harder* drugs [6]. Moreover, according to a study by McCarthy [10], 1 in 8 Americans report smoking marijuana [10]. This suggests that the current interventions aiming to prevent substance use escalation targeted at those who use marijuana may not be sufficiently granular. Furthermore, much of the research to date is either cross-sectional or focused on a single substance rather than across substances with variance in perceived risk. To fill this gap, our study aimed to investigate substance use trajectories on social media to improve our understanding of transitions between types of substances, factors associated with the likelihood of progression to higher-risk substances, and the linguistic patterns associated with escalating and de-escalating.

Social media platforms such as X (previously known as Twitter), Reddit, and YouTube have created spaces where individuals can engage with each other and share various parts of their private lives, including their personal experiences and life events [11-13]. This is especially true among individuals who misuse substances, as networking with others with lived experiences has been shown to decrease self-stigma and encourage safer substance use behaviors and recovery efforts [14]. However, it should be noted that the increased visibility and influence of these platforms might inadvertently normalize substance misuse and provide easier access to information related to harmful substances, thus potentially promoting harmful substance misusing behaviors [15,16]. Nevertheless, the data from social media are invaluable for research. The information gathered from social media platforms is timely and relevant for studying substance use characteristics longitudinally [17-19]. As a result, the data obtained from social media offer a unique opportunity to observe substance use behaviors within a broad population, enabling a deeper comprehension of the underlying patterns and factors influencing substance use behaviors.

Several studies have used social media data to examine different aspects of substance-related behaviors, such as adverse drug reactions [20,21], substance misuse [22,23], and substance use–related slang [24]. Lu et al [25] used machine learning techniques to estimate Reddit users’ shifts from casual drug discussion forums to drug recovery forums. They further developed a survival model to estimate the probability of a Reddit member posting in a recovery forum within the upcoming year [26]. Another work developed a language model of opioid consumption and generated alternative words for opioids, routes of administration, and drug tampering [27]. One study [28] used transfer learning techniques to detect Reddit posts that indicate opioid recovery. With a combination of deep learning and human annotation techniques, they identified and analyzed the effectiveness of particular alternative drugs for treating opioid use disorder (eg, kratom). Relatedly, another study [29] used social media content related to the misuse of fentanyl to build a machine learning model to identify risky discourse and develop a vocabulary set consisting of community-specific and colloquial terms associated with fentanyl and its analogs [29]. However, few studies have assessed the gateway hypothesis using social media data. Furthermore, to our knowledge, none of the existing studies have focused on identifying or understanding specific risky behaviors associated with the gateway hypothesis.

### Study Objectives

In this work, we investigated substance use behaviors on social media, particularly the transitions between substances with different risk levels. We specifically addressed the following research questions (RQs):

- RQ1: Does substance use–related social media behavior support the gateway hypothesis?
- RQ2: How can we use social media to identify transitions in different risk levels of substances?
- RQ3: What specific linguistic cues in web-based behaviors can be identified using social media data to distinguish between escalating and de-escalating risk transitions?

To answer these RQs, we analyzed Reddit data to understand the trajectory of substance use: a Reddit user’s entire historical path of posts and comments within targeted substance-related subreddits, encoded by risk levels assigned to these subreddits. This risk level is a numeric indicator assigned to each subreddit based on the potential harm of the substance use behaviors discussed, ranging from discussions of nonharmful substances to those with a high potential for those with a high potential for substance use disorder and harm. We then examined *transitions*, defined as changes in risk levels from one time bin to another within a user’s trajectory, to identify potential early signs of risk level changes associated with substance use. A transition begins when we observe a series of time bins (groupings of weekly posts and comments) that have the same risk level. The transition is marked when a subsequent time bin displays a different risk level, indicating a change in the Reddit user’s posting and commenting with either a higher-risk (escalating transition) or a lower-risk (de-escalating transition) substance-related subreddit. This shift from one risk level to another defines the transition, capturing the Reddit user’s movement toward potentially different substance use behaviors. We implemented a logistic regression model, a Bidirectional Encoder Representations from Transformers (BERT) model, and a Robustly Optimized BERT Approach (RoBERTa) model to identify potential escalation or de-escalation transition in risk levels. We show the potential of using deep learning methods with large-scale historical data to predict the escalation or de-escalation transitions of risk levels. Our linguistic analyses showed the most salient linguistic cues between escalating transitions and de-escalating transitions. These findings highlight the importance of language patterns within self-generated content related to substance use trajectories. They suggest the need for further research to refine these methodologies and the future applications to use social media data to analyze substance use behaviors to refine policies and therapeutic strategies targeting substance use disorders and recovery strategies.

## Methods

### Data Source

Past research has suggested that Reddit is a popular social media platform well suited for substance-related research due to its large number of users with diverse backgrounds, which facilitates longitudinal data analysis and insights into the evolving nature of substance use and discussions [30]. Various studies have indicated that engaging in substance-related discussions can be a strong marker of an individual’s experience with substance use [26,28,31]. For our data set, we focus on Reddit, a prevalent social media platform where individuals can anonymously participate in topic-centered communities known as “subreddits.” Each subreddit operates under its own set of rules to moderate posts and comments, offering unfiltered discussions with less interference from overarching platform policies, distinguishing Reddit as an ideal platform for analysis of substance-related conversations [30]. On Reddit, there are various subreddits dedicated to discussing different substances, such as cannabis, cocaine, and fentanyl, as well as behaviors associated with these substances, such as active misuse, harm reduction, and recovery.

Grounding our approach in psychiatric theory related to the developmental patterns of substance involvements [8,19,32-35] and the discussions within these substance-related subreddits, we curated a list of relevant subreddits and annotated each subreddit based on the risk level associated with specific substances discussed. The rationale behind annotating at the subreddit level rather than the post level is the structural organization of Reddit itself. Subreddits are often named after or centered on a specific topic, suggesting that a majority of posts within a given subreddit, such as *r/fentanyl*, likely align with a particular risk profile related to fentanyl. Furthermore, subreddits are typically guided by a set of rules and guidelines enforced by moderators to ensure that the content remains relevant to the subreddit’s central theme. The presence of these regulations provides a level of consistency in the nature and risk associated with the content, making it more practical to assess and annotate at the subreddit level.

To create a comprehensive list of relevant subreddits, we used a methodology similar to the previous research [36]. We began by compiling an extensive lexicon of terms related to substance use, focusing on keywords that encompass various facets of the experience and treatment of substance use disorders. This included a range of drug names, incorporating substances available over the counter or through prescription or those considered illicit. For every generic drug name, we also incorporated the corresponding trade names and combination products, drawing upon insights from substance use research and from collaboration with coauthors specializing in clinical psychology. We found 152 keywords in this process. We then used these 152 keywords to query Reddit posts through BigQuery (Google). Our focus was not limited to the most popular or active subreddits; instead, we aimed to encompass a diverse representation of the substance use spectrum, including active use, harm reduction, and recovery. This process led to the curation of 73 drug-related subreddits.

Using a set of 73 drug-related subreddits, we collected all the posts and comments from Google Big Query [37]. Google Big Query is the publicly available data warehouse containing all the posts and comments made on Reddit. We used Google’s BigQuery application programing interface to query publicly available Reddit data from December 2015 to December 2019. With increasing privacy concerns and changes in data protection laws, researchers frequently find themselves limited to using dated historical data to gain insights from social media platforms. In the *Discussion* section, we revisit the limitation of the temporal restriction of our data set. In total, we collected 2,291,356 posts and 29,372,044 comments created by 1,426,621 Reddit users. We excluded 4321 authors with usernames beginning or ending with “bot” and deleted accounts. We also removed those with <1 year of posting and commenting activities in our collected data set to ensure we had enough historical data to conduct the analysis. After these steps, 313,846 authors remained in our data set.

### Ethical Considerations

Although we use publicly accessible historical Reddit data from Google BigQuery that do not qualify for ethics board approval, we are committed to protecting the privacy of the data owners. Following the prior work on sensitive topics on social media [28,38,39], we have removed any personally identifiable information and have paraphrased all quotations in this paper to avoid traceability. The authors of this paper have backgrounds in both computer science and data analytics, as well as clinical psychology and addiction medicine. Such a diverse knowledge base enhances our understanding of the privacy considerations of this group and allows an interdisciplinary approach to understanding our findings.

### Annotation Approach

The coauthors in this study, who are substance use domain experts, used inductive and deductive approaches to develop a risk level codebook based on substance type. First, substance use literature was reviewed, and a rough structure was developed for the codebook based on respective risks related to each substance (ie, the risk for overdose, dependence, risky routes of administration, etc). Next, coders systematically examined each of the 73 subreddits in our data set to understand the substances and the level of risk discussed in each community and to refine the categories in the codebook. This process involved 2 independent coders (Kevin Davet and Nina Kaiser) searching the subreddit name on Reddit, reviewing the first 25 posts and their associated comments listed in each subreddit, and then assigning a risk category to this subreddit based on the substance and substance use behaviors mentioned in this community. Interrater reliability was 96% for the initial risk category assigned by the 2 independent coders, and the remaining discrepancies were discussed among a group and resolved by a third coder (EK). Once a final codebook was established, coders worked to assign a risk level to each subreddit ranging from 0 to 4, with 0 denoting no risk and 4 indicating of the highest level of risk. Multimedia Appendix 1 [40-49] provides the details on risk level definitions by substance used in this study.

The domain expert coauthors determined that 1 subreddit in our data set (*r/supplements*) did not constitute a risky substance subreddit. This was the only subreddit that was assigned to level 0. Due to the underrepresentation of the risk level 0, we excluded the subreddit (*r/supplements*) from our subsequent analysis. We also excluded 7 subreddits annotated as *risk level mixed* (*r/drugtesthelp, r/DrugNerds, r/REDDITORSINRECOVERY, r/addiction, r/ReagentTesting, r/Drugs, r/drugsarebeautiful*). These subreddits discussed multiple substances and different aspects of substance use behaviors that were placed in the high-risk category. In the end, we had the following data set for the subsequent analysis: risk level 1 had 3 subreddits, risk level 2 had 22 subreddits, risk level 3 had 8 subreddits, and risk level 4 had 32 subreddits (Textbox 1). Among these subreddits, we had 133,220 Reddit users with 9,205,558 posts and comments.

**Categorization of subreddits according to risk levels.**

**Categorization**

- Risk level 0: *r/Supplements*
- Risk level 1: *r/SMARTRecovery, r/secularsobriety, r/AtheistTwelveSteppers*
- Risk level 2: *r/TokeSpot, r/vaporents, r/CannabisExtracts, r/EntExchange, r/Kanna, r/GetOutAndVape, r/quittingkratom, r/AthleticEnts, r/kratom, r/cannabis, r/mod quittingkratom, r/trees, r/Petioles, r/leaves, r/entshop, r/portabledabs, r/abv, r/Waxpen, r/vapebonging, r/StonerEngineering, r/eldertrees, r/treedibles*
- Risk level 3: *r/researchchemicals, r/Tianeptine, r/tryptonaut, r/TripSit, r/pharms, r/Psychonaut, r/Nootropics, r/afinil*
- Risk level 4: *r/OpiatesRecovery, r/HeroinHeroines, r/MDMA, r/lean, r/naltrexone, r/heroin, r/suboxone, r/gabapentin, r/opiates, r/opiatesmemorial, r/ObscureDrugs, r/phenibut, r/pillhead, r/pregabalin, r/loperamide, r/Opiatewithdrawal, r/meth, r/Methadone, r/askdrugs, r/fentanyl, r/drugwar, r/benzodiazepines, r/stimcirclejerk, r/Stims, r/heroinaddiction, r/benzorecovery, r/oxycodone, r/OurOverUsedVeins, r/OpiateChurch, r/Thewarondrugs, r/drugscirclejerk, r/Carfentanil*
- Risk level mixed: *r/drugtesthelp, r/DrugNerds, r/REDDITORSINRECOVERY, r/addiction, r/ReagentTesting, r/Drugs, r/drugsarebeautifu*

### Approach Overview

Our study aimed to investigate the gateway hypothesis on Reddit through 3 RQs: analyzing social media behaviors that support the gateway hypothesis (RQ1), predicting transitions between substances (RQ2), and comparing the linguistic differences between escalating and de-escalating risk transitions (RQ3). To tackle these RQs, we began by categorizing Reddit users into groups based on their historical trajectories and analyzed their behaviors to answer RQ1. We then constructed a risk transition data set using the trajectories of Reddit users from our collected data sets. For RQ2, we used the risk transition data set to develop and compare different machine learning and deep learning models for forecasting the future directions of risk transitions. In addition, we extracted coefficient scores for each input feature from our machine learning models to identify the most important features in distinguishing between future escalating and de-escalating transitions. To address RQ3, we used an unsupervised language modeling technique to measure the linguistic disparities in posts and comments created on initial transition phases between escalating and de-escalating transitions.

### Modeling Trajectories and Transitions

#### Overview

To evaluate the gateway hypothesis on Reddit, we investigated and analyzed the historical activity of every Reddit user in our collected data set. Each Reddit user’s posts or comments history with substance-related subreddits was compiled into a single trajectory. Our approach involved constructing a trajectory for each Reddit user in our data set, encompassing all the posts and comments they had made across 65 substance-related subreddits. This enabled us to investigate whether a pattern emerged among Reddit users, beginning with discussions or interactions related to specific substances and subsequently transitioning toward others.

For each Reddit user’s trajectory, we replaced the subreddit names that a Reddit user had posted or commented on with the annotated risk levels of that subreddit as assigned by our domain experts. By broadening our analysis beyond specific substances and focusing on risk levels, we aimed to explore Reddit users’ overall progression and movement across different levels of risk within the substance-related subreddit landscape.

#### Time Binning

We grouped posts and comments into bins by each active week to reduce noise in our data. This grouping process involved defining a starting time, denoted as *t*, which represents the time stamp of the first post or comment created. We then established a week-long time frame by adding the total number of seconds in a week to *t*. Thus, an active week bin encompasses all posts and comments with time stamps falling within the range of *t* *to* *t*+(number of seconds in a week). To establish the subsequent active week, we identified the time stamp *t* of the next post or comment that occurs after *t*+(number of seconds in a week). We assigned a majority label within each bin based on the posts and comments in the bin. This involved computing the risk label for the bin by converting the subreddits present within it into their respective risk levels. We then determined the risk level that occurred most frequently within the bin, establishing the majority label for that particular period.

#### Breaking Ties

Following the process of creating time bins for each trajectory and assigning majority labels to those bins, we found that approximately 5% of Reddit users in our data set encountered ties in their posting or commenting history at the subreddit level. A tie occurs when multiple labels have the same frequency of occurrence within a given time bin. In such cases, we used the strategy of breaking ties by selecting the highest risk level as the majority label. The rationale behind this tie-breaking approach lies in the assumption that when Reddit users begin engaging in discussions within higher risk level subreddits, they likely already possess some level of experience or familiarity with those particular drugs [26,28,31]. By assigning the higher risk level as the majority label in cases of ties, we aimed to capture the progression or transition of Reddit users toward subreddits associated with potentially greater risk. Furthermore, we discussed more about the limitation of this approach in assigning majority labels in the *Discussion* section, acknowledging the complexities and nuances involved in such categorizations.

#### Grouping Trajectories

To understand how Reddit users exhibit different behaviors within their trajectories, we grouped each trajectory into three classes; these classes allowed us to categorize trajectories based on the patterns observed in their risk levels over time. (1) *Escalating trajectories*: this class includes trajectories demonstrating a clear progression or escalation in risk levels across the time bins within the trajectories. It indicates a pattern where Reddit users gradually shift from lower-risk subreddits to those associated with higher risk levels. (2) *De-escalating trajectories*: in contrast to escalating trajectories, de-escalating trajectories exhibit a pattern of decreasing risk levels over time. (3) *Trajectories with no change*: trajectories falling into this class consist of time bins labeled with the same risk level throughout.

#### Creating Transitions

After transforming each Reddit user’s activity into one continuous trajectory by applying time binning and resolving ties, we analyzed the data by creating transitions from these trajectories. While each Reddit user in our data set has one trajectory, within this trajectory, a Reddit user may experience *multiple transitions* that is defined as shifts from one or multiple consecutive time bins with the same risk level to a time bin with a different risk level.

**Figure 1:**
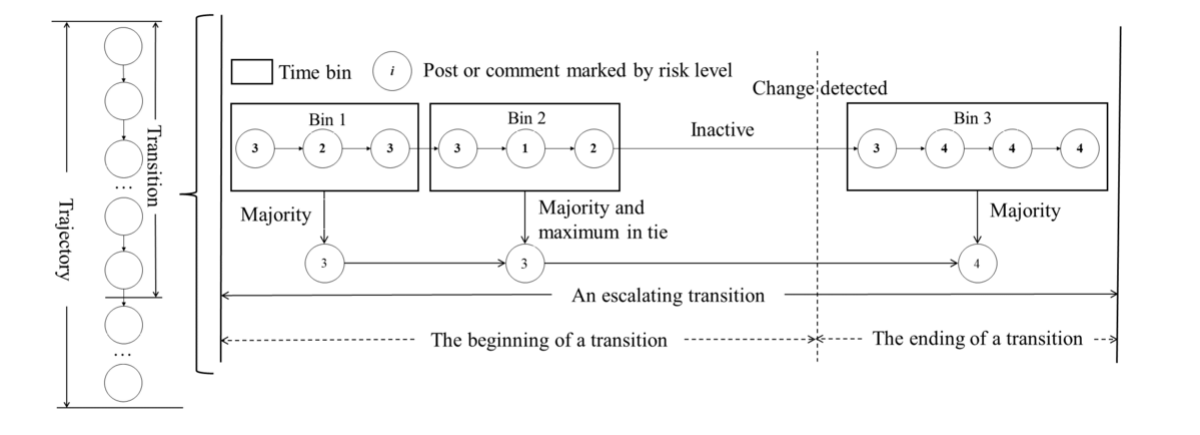
A flowchart showing risk transition across 3 time bins. A transition within a trajectory represents a change in the risk level from one time bin to the next. The beginning of a transition is indicated by a series of consecutive time bins with the same risk level, followed by a time bin with a different risk level. Each bin evaluates posts or comments marked with risk levels, depicted by numbered circles. Bin 1 and bin 3 use majority voting, while bin 2 considers both majority and maximum values in case of a tie.

To identify these transitions, we first located the beginning of a transition (Figure 1). This occurs when a time bin with the same risk level persists consecutively before encountering a time bin with a different risk level. The beginning of a transition signifies the period where the Reddit user’s activity remains within a consistent risk level. Subsequently, the ending of a transition marks the point where the risk level changes within the trajectory, represented by a single time bin with a different risk level than the preceding bins. Once an ending transition is identified, the subsequent beginning transition starts with the time bin following the ending transition. This approach allows us to delineate the risk transitions within a Reddit user’s trajectory, capturing the shifts in risk levels and potentially indicating the *escalation transitions* or *de-escalation transitions* in their engagement with substance-related subreddits. We removed all the transitions that contained <100 words at the beginning of the transitions to ensure that we had enough data to conduct the following analyses.

### Predicting the Future Risk Transitions

#### Overview

For prediction, we used a logistic regression model, a popular interpretable machine learning model, to perform text classification tasks. Using transformer-based pretrained models has become the norm in various natural language processing fields and has achieved state-of-the-art performances [50]. Motivated by this, we used BERT [51] and its extension, RoBERTa [52], as additional methods.

#### Logistic Regression Model

The logistic regression model was developed to predict the direction of transitions between subreddit risk levels by using a binary dependent variable indicative of the transition direction (escalating transitions vs de-escalating transitions). Independent variables, or predictors, were derived from textual features and user engagement metrics extracted at the beginning of identified transitions. Four categories of predictors were extracted from the model:

1. Psycholinguistic attributes: the Linguistic Inquiry and Word Count (LIWC) lexicon [53-55] is a well-validated psycholinguistic lexicon used for extracting psycholinguistic characteristics from texts. It analyzes texts across various dimensions, such as emotions, perceptions, interpersonal focus, and social and personal concerns. For each transition, we used the LIWC lexicon to obtain normalized occurrences of words in each LIWC category for each individual.
2. Open vocabulary (n-grams): the concept of open vocabulary, which refers to the unrestricted and diverse set of words or word combinations a person can use, has been used to investigate the psychological attributes of individuals in multiple previous studies [38,56,57]. We calculated the distribution of the top 500 uni- and bigrams at the beginning of the transition as open vocabulary features.
3. Part-of-speech tagging: the Stanford Log-linear Part-Of-Speech Tagger [58] was used to identify nouns, verbs, and adjectives in posts and comments. This tool calculates the ratios of nouns, verbs, and adjective words in the posts and comments.
4. Activities: for each Reddit user in our collected data set, we measured the average number of Reddit posts or comments made within our data set per bin and the number of words per bin.

After extracting these 4 sets of features from the posts and comments made during the initial stages of the transition, we constructed a logistic regression model. This model predicts whether an individual’s transition is escalating or de-escalating. It considers the independent variables, in this case, the extracted features: psycholinguistic attributes, open vocabulary, part-of-speech tagging, and activities, and outputs a binary prediction of whether the transition will escalate or de-escalate in the future. To ensure the optimal performance of our model and reduce the noisy features, we applied a feature selection strategy, determining to retain the top 100 features that exhibit the highest levels of mutual information with the classification output. This approach allowed us to focus on the most critical features and facilitate a more effective predictive model.

We randomly split our entire transitions data set into training and testing sets in an 8:2 ratio to evaluate our logistic regression model. During the training phase of the model, we used cross-validation to ensure that our model generalizes well to unseen data and does not overfit. Specifically, we adopted a -fold cross-validation approach, where is set to 5. The performance across all iterations was then averaged to provide a more accurate measure of the model’s performance.

#### Deep Learning Model

To tackle the multifaceted problem of predicting future risk levels for each substance-related transition, we used BERT [51] and its extension, RoBERTa [52]. As a pioneer in transformer-based models, BERT revolutionized the natural language processing field with its bidirectional training approach. RoBERTa further improved upon it by modifying key hyperparameters in the model architecture, such as removing the next-sentence pretraining objective and training with much larger minibatches [52]. Both models have been adapted to solve various domain-specific tasks, such as suicidal ideation [53], mental disorders detection [59], web-based social support [60], and social dimensions describing human relationships [61].

We fine-tuned pretrained BERT and RoBERTa models using concatenated texts at the beginning of transitions as inputs and the corresponding escalation directions as outputs. To assess the performance of the fine-tuned models, we randomly split the entire transition data set into training and testing sets in an 8:2 ratio and used a *k*-fold cross-validation (*=5*) approach on the training data set. In our training setting, we set the epoch count to 5, a decision guided by initial tests that observed marginal improvement of the model beyond this epoch number. We set the batch size to 32, considering our computational capacity. The learning rate was chosen as 5 ×10^−5^ by default.

### Analyzing the Linguistic Cues

We used an unsupervised language modeling technique known as the Sparse Additive Generative Model (SAGE) [62] to investigate the linguistic indicators at the concatenated texts created during the beginning phase of escalating or de-escalating transitions. This approach enabled us to compare the parameters of 2 multinomial models with the logistic regression, allowing us to identify significant terms. The regularization parameter in SAGE was self-tuned, and this balanced the importance of common and rare terms in the selection process. We applied the SAGE technique specifically to differentiate between n-grams (n=2, 3) present in the posts and comments at the beginning of transitions between escalating and de-escalating transitions.

During our analysis, a positive SAGE value (>0) suggested that an n-gram was more representative of escalating transitions, indicating its association with an increase in risk levels. Conversely, a negative SAGE value suggested greater representativeness for the absence of escalating transitions, indicating its association with a decrease in risk levels. By examining these linguistic indicators using the SAGE technique, we gained insights into the specific language patterns and characteristics that distinguished escalating and de-escalating transitions within our data set. This analysis helped us better understand the linguistic dynamics and factors that influenced the progression or regression of risk levels on Reddit.

## Results

### Modeling Trajectories and Transitions

Table 1 provides an overview of the activity of Reddit users within drug-related subreddits, highlighting the number of Reddit users, the frequency of posts and comments, and the instances of risk level transitions. A subset of users displayed at least 1 transition in risk level behavior, with variations in the length and frequency of their posts and comments. These data show the analysis of transitions between subreddits of differing risk levels (Table 1).

**Table 1:** Summary of Reddit users across 65 drug-related subreddits, their post and comments, and transitions statistics.

Variable			Values
Reddit users, n			133,220
Reddit users who have at least 1 transition, n (%)			58,279 (43.75)
Posts and comments, n			9,205,558
Transitions, n			169,811
**Posts and comments length (by words)**			
	Mean (SD)	36.67 (65.49)	
	Median (range)	18 (1-9688)	
**Subreddits that Reddit users posted or commented**			
	Mean (SD)	2.69 (2.00)	
	Median (range)	2 (1-48)	
**Unique risk levels per Reddit users**			
	Mean (SD)	1.67 (0.68)	
	Median (range)	2 (1-4)	
**Posts and comments per Reddit user**			
	Mean (SD)	69.10 (280.91)	
	Median (range)	15 (1-36,533)	
**Posts and comments at the beginning of transition**			
	Mean (SD)	18.12 (97.58)	
	Median (range)	3 (1-6650)	

Table 2 presents the number of Reddit users based on their historical trajectories in our collected data set, specifically their risk transition patterns. The table categorizes Reddit users into 3 types based on their trajectories: escalation trajectories, de-escalation trajectories, and no change. For each category, the table shows the starting and ending risk levels of the transition within trajectories, the total number of users who exhibited the corresponding transition in their trajectories in our collected data set, and the average transition time (ATT) duration in weeks. Note that no ATT is recorded for users showing no change in risk levels.

**Table 2:** Distribution of Reddit users categorized by their historical behavior patterns^a^.

Trajectories type and users, n (%)			Risk level transition				ATT^b^ (weeks), mean (SD; range)
			Start		End		
**No change (n=39,000)**							
	26,381 (67.6)	2		2		—^c^	
	7814 (20)	4		4		—	
	4805 (12.3)	3		3		—	
**Escalation (n=8,428)**							
	3706 (44)	2		4		39.08 (42.55; 1.00-240.30)	
	3617 (42.9)	2		3		39.71 (42.30; 1.00-224.04)	
	1105 (13.1)	3		4		28.71 (35.97; 1.02-220.40)	
**De-escalation (n=4925)**							
	2065 (41.9)	4		2		32.73 (36.48; 1.00-231.16)	
	1936 (39.3)	3		2		35.79 (38.36; 1.00-227.81)	
	924 (18.8)	4		3		29.51 (35.93; 1.00-210.12)	

^a^Note that no average transition time is recorded for Reddit users, showing no change in risk levels.

^b^ATT: average transition time.

^c^Not available.

Regarding Reddit users with trajectories indicating only the escalation in risk levels across the time bins, Reddit users predominantly showed transitions from risk level 2 to 4, followed by transitions from 2 to 3 and 3 to 4. The chi-square test for goodness of fit on the number of Reddit users in escalation trajectories showed significant differences (*χ*^2^_3_=1552.4, *P*<.001). For example, one Reddit user wrote about their first-time kratom (risk level 2) experience in the risk level 2 subreddit*:*

Yesterday I had my first experience taking Kratom...

After around 39 weeks, the same user wrote another post disclosing their own experiences with phenibut (risk level 3), varying dosages, frequency, and the effects of combining it with other substances:

I’ve been using phenibut ranging from once every 2 weeks to 3 times a week...I took 2g followed by 2, 1g redoses...I took 2g early on the day and decided to drink like 3 beers probably 12 hours later.

The ATT for these transitions ranged from 28.71 (from risk level 3 to risk level 4) to 39.71 weeks (from risk level 2 to risk level 3). In the group with de-escalating trajectories, Reddit users primarily showed transitions from risk level 4 to 2 (*χ*^2^_3_=475.7, *P*<.001), with the ATT being 32.73 weeks. Transitions from 3 to 2 and 4 to 3 also occurred, with average active use ranging from 29.51 to 35.79 weeks. For the group with no-change trajectories, most Reddit users in our data set remained at risk level 2 without any transition as indicated by a significant chi-square result. (χ^2^_3_*=*21,008.0, *P*<.001).

### Predicting the Future Risk Transitions

**Figure 2:**
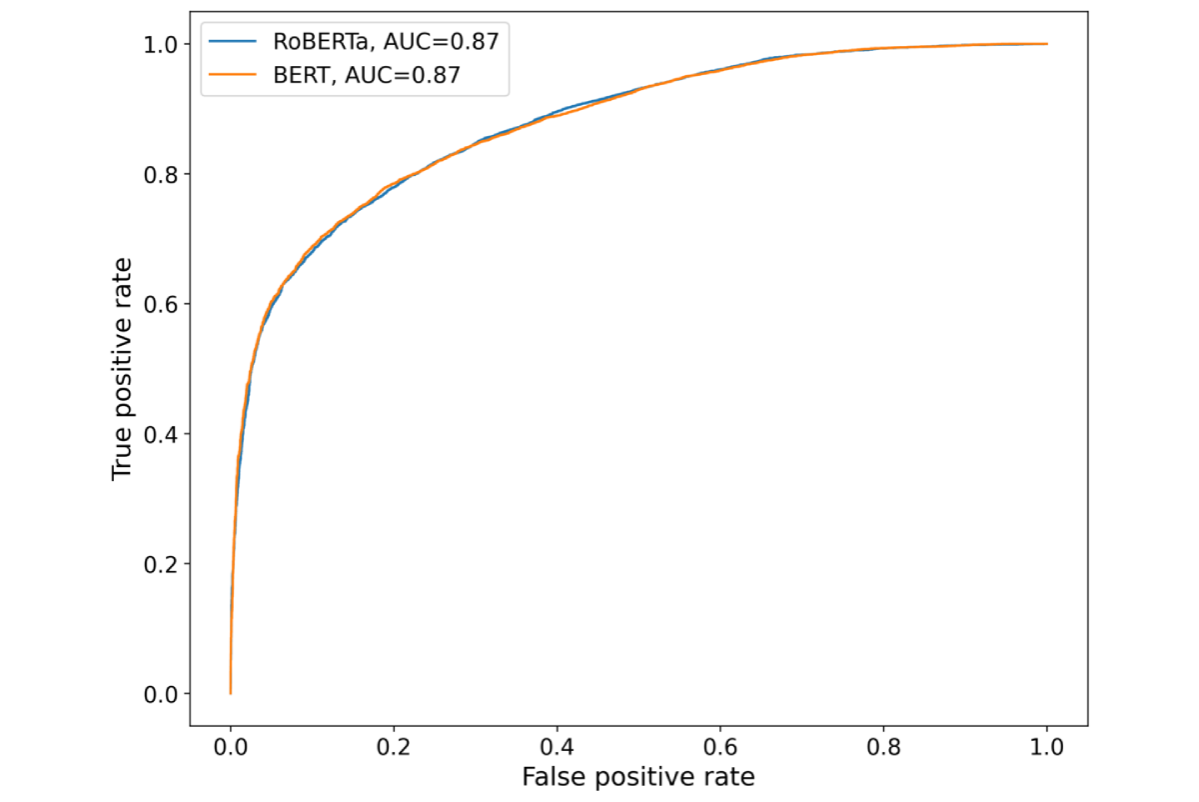
Receiver operating characteristic curves comparing the performance of Bidirectional Encoder Representations from Transformers (BERT) and Robustly Optimized BERT Approach (RoBERTa) models on a test data set. AUC: area under the curve.

We implemented and evaluated multiple classifiers on our transition data set to predict the future transitions of risk level, including the logistic regression, BERT, and RoBERTa models. Table 3 summarizes the performance of our models and includes the accuracy, precision, recall, *F*_1_-score, and area under receiver operating characteristic curve score (Figure 2). These metrics are critical for evaluating the performance of machine learning and deep learning models [63,64]. On the basis of these results, we found that the fine-tuned BERT and RoBERTa models had better performance than the logistic regression model. The BERT model, after fine-tuning, achieved an accuracy of 78.48% and an *F*_1_-score of 79.2%, while logistic regression had an accuracy of 75.5% and an *F*_1_-score of 76.08%. The differences in their performance might suggest the complex language patterns found in substance use–related discussions. Overall, these models showed promise in predicting the future risk level, with the fine-tuned BERT and RoBERTa models demonstrating better performance than logistic regression.

**Table 3:** Comparative performance metrics of logistic regression, Bidirectional Encoder Representations from Transformers (BERT), and Robustly Optimized BERT Approach (RoBERTa) models on a 5-fold cross-validation and test data set.

Model		Accuracy	Precision		Recall	*F*_1_-score	ROC-AUC^a^
**5-fold cross-validation, mean (SD)**							
	Logistic regression	75.29 (0.29)	80.55 (0.30)		70.97 (0.39)	75.45 (0.29)	81.99 (0.29)
	BERT	79.14 (0.27)	82.34 (1.96)		77.85 (2.25)	79.98 (0.75)	87.93 (0.37)
	RoBERTa	79.09 (0.53)	84.97 (1.15)		74.13 (2.68)	79.14 (1.04)	87.86 (0.52)
**Test data set (%)**							
	Logistic regression	75.51	80.92	71.78		76.08	82.34
	BERT	78.48	82.84	75.87		79.2	87.29
	RoBERTa	78.28	84.26	73.51		78.52	87.09

^a^ROC-AUC: area under the receiver operating characteristic curve.

Although the BERT and RoBERTa models showed better performance metrics than the logistic regression model, it is crucial to recognize the interpretability and explainability of the logistic regression model. As an explainable model, logistic regression allows for the extraction and examination of the most influential features in its decision-making process [65]. Table 4 shows the top 20 predictive features (independent variables) with the β coefficients and significance from the logistic regression. A subreddit with a positive β coefficient indicates that the occurrence of the features in the posts increases its possibility of future escalation into a higher risk level subreddit (escalating transition from a lower risk level subreddit to a higher risk level). A subreddit with a negative β coefficient indicates that the occurrence of the features in the posts increases its possibility of future de-escalating into a lower risk level subreddit (de-escalating transition from a higher risk level subreddit to a lower risk level). In the predictors associated with future risk escalations, we found many n-gram features related to substance names, including *kratom*, *weeds*, *marijuana*, *tree*, *buds*, and *cannabis*, showing high relative importance. We also observed words related to substance-related behaviors or tools, such as *bong*, *vape*, *smoke, smoking, *and *smoked*, showing high relative importance.

**Table 4:** Top 20 features in the logistic regression classifier with the β coefficients. *P* values reported after Bonferroni correction. Positive features indicate the most predictive features for identifying escalating transitions, and negative features suggest the most predictive features for identifying de-escalating transitions.

Feature			β		*P* value
**Positive**					
	kratom	.510		<.001	
	bong	.261		<.001	
	weed	.249		<.001	
	smoke	.246		<.001	
	smoking	.216		<.001	
	marijuana	.214		<.001	
	vape	.202		<.001	
	bud	.190		<.001	
	joint	.179		<.001	
	strain	.167		<.001	
	bowl	.166		<.001	
	edible	.158		<.001	
	tree	.156		<.001	
	oil	.130		<.001	
	high	.123		<.001	
	cannabis	.099		<.001	
	legal	.095		<.001	
	glass	.094		<.001	
	green	.087		<.001	
	smoked	.084		<.001	
**Negative**					
	phenibut	−.351		<.001	
	mdma	−.265		<.001	
	pill	−.258		<.001	
	xanax	−.234		<.001	
	meth	−.225		<.001	
	opiate	−.220		<.001	
	roll	−.191		<.001	
	LlWC^a^:bio	−.138		<.001	
	LlWC:insight	−.111		<.001	
	heroin	−.110		<.001	
	trip	−.105		<.001	
	LlWC:time	−.097		<.001	
	take	−.082		<.001	
	benzos	−.081		<.001	
	LlWC:you	−.079		<.001	
	LlWC:death	−.075		<.001	
	shit	−.064		<.001	
	took	−.063		<.001	
	mg	−.062		<.001	
	experience	−.060		<.001	

^a^LIWC: Linguistic Inquiry and Word Count.

In n-gram features associated with future risk de-escalation transitions, the n-gram features indicated possible treatments, such as *phenibut*, *pills*, and *xanax.* We also found that the n-gram features around “harder substances” are reported to have high relative importance in predicting future risk de-escalation transitions such as *mdma*, *meth*, *heroin*, and *opiate*. LIWC features, including biological processes, insight, time, you (second pronouns), and death, contributed significantly to predicting future risk de-escalation transitions. This might be because Reddit users used these words to describe their symptoms or give suggestions or warnings at the beginning of their de-escalating transitions.

### Analyzing the Linguistic Cues

To investigate the most salient linguistic cues at the beginning of escalating and de-escalating transitions, we adopted the SAGE analysis [62] and reported the top salient n-grams in Table 5. A positive SAGE score indicates the terms frequently associated with the beginning of escalation transitions, while a negative SAGE score points to the terms linked with the beginning of de-escalation transitions.

We discovered that keywords such as *taking kratom*, *smoking weed*, and *dry* *herb* related to substance use frequently appear in individuals’ self-motivated texts before transitioning into higher-risk subreddits. For instance, one person described their experiences with kratom:

I was taking kratom for over a year or two and had positive experiences. However...

Approximately 31 weeks later, the same author wrote in a risk level 4 subreddit:

I was in a temporary role, and they offered me a full-time position, contingent on passing a drug test. I usually don’t consume opiates, but I took 6 pills on December 24th...

We noted that keywords describing substance use; purchase locations; and feelings associated with use, such as *head shop*, *smell like*, and *get high*, were more salient in the language of escalating transitions (eg, “I went to the *head shop*,” “My room *smells like* cannabis,” and “I *get high* from it.”).

In contrast, keywords indicating harm reduction and recovery, such as *test kit*, *get clean*, and *stay safe* were prevalent at the beginning of de-escalating transitions. We observed these keywords frequently in texts describing individuals’ recovery experiences from substance misuse and providing suggestions for others. For example, a post reads as follows:

To get clean, I reduced my intake gradually over two months, decreasing my dosage by 25% at each step.

Roughly 20 weeks later, the same poster sought suggestions and support in a lower-risk subreddit regarding severe withdrawal symptoms:

I used to struggle with anxiety...and a strong desire for Xanax. While many of these symptoms have subsided, I often find myself without an appetite...I am inquiring whether Xanax withdrawal can lead to this issue.

We also observed many time-related keywords with high salience scores in de-escalating transitions, such as *3 months*, *hour late*, *year clean*, and *3 days*. This suggests that before posting or commenting on lower-risk subreddits, Reddit users might have previously shared their experiences on higher-risk subreddits. Additionally, keywords suggesting reduced substance use, such as *take half*, *half pill*, and *extended release*, appeared more frequently in de-escalating transitions. This might indicate that individuals transitioning to lower-risk subreddits are sharing their harm reduction strategies and experiences that involve reducing the amount of substance consumed.

**Table 5:** Top salient n-grams (n=2, 3) associated with escalating and de-escalating transitions in posts, as identified by Sparse Additive Generative Model (SAGE) analysis. A positive SAGE score indicates the terms frequently associated with the beginning of escalation transitions, while a negative SAGE score points to the terms linked with the beginning of de-escalation transitions.

n-gram			SAGE
**Top salient n-grams in escalating transitions**			
	legal, state	0.155	
	taking, kratom	0.144	
	look, like	0.141	
	illegal, state	0.141	
	coconut, oil	0.136	
	started, smoking	0.134	
	smoking, weed	0.128	
	maeng, da	0.119	
	using, kratom	0.106	
	smoke, spot	0.101	
	dry, herb	0.090	
	different, strain	0.087	
	mason, jar	0.086	
	green, malay	0.086	
	like, weed	0.080	
	stop, smoking	0.079	
	red, bali	0.078	
	medical, marijuana	0.078	
	want, smoke	0.076	
	take, kratom	0.075	
	head, shop	0.073	
	people, smoke	0.072	
	smell, like	0.072	
	tolerance, break	0.072	
	peanut, butter	0.071	
	get, high	0.071	
	smoke, weed	0.069	
	kratom, use	0.068	
	pax, 2	0.067	
	drug, test	0.066	
	plant, matter	0.065	
	time, day	0.064	
	arizer, air	0.063	
	fellow, ents	0.061	
	smoke, much	0.060	
	one, hitter	0.060	
**Top salient n-grams in de-escalating transitions**			
	test, kit	−0.130	
	get, clean	−0.093	
	stay, safe	−0.089	
	first, time	−0.077	
	harm, reduction	−0.076	
	3, month	−0.069	
	half, life	−0.058	
	take, mdma	−0.057	
	stay, clean	−0.056	
	hour, later	−0.055	
	taking, phenibut	−0.054	
	getting, clean	−0.052	
	sound, like	−0.052	
	third, eye	−0.051	
	take, phenibut	−0.050	
	pressed, pill	−0.049	
	take, half	−0.049	
	3, day	−0.049	
	happy, nod	−0.048	
	rebound, anxiety	−0.047	
	never, done	−0.047	
	day, row	−0.047	
	taking, mdma	−0.046	
	poppy, seed	−0.045	
	got, clean	−0.045	
	extended, release	−0.044	
	drug, use	−0.044	
	pure, mdma	−0.044	
	half, pill	−0.043	
	first, roll	−0.043	
	would, take	−0.042	
	methadone, clinic	−0.042	
	year, clean	−0.042	
	xanax, bar	−0.042	
	next, day	−0.041	
	get, script	−0.041	

## Discussion

### Principal Findings

Overall, the results of this study analyzing substance use–related social media behavior on Reddit show preliminary support for the gateway hypothesis; however, these results were mixed and require greater contextualization and examination; that is, while some of the proponents of the gateway hypothesis were supported based on patterns of escalation trajectories (marijuana subreddits to higher-risk subreddits), other patterns noted for escalation and de-escalation trajectories were more complex and were not supported by the original gateway framework. For example, we did observe that engagement in marijuana-related subreddits along with the pattern of specific n-grams such as *kratom*, *weeds*, and *marijuana* were predictive of future risk escalation transitions. This aligned with the gateway hypothesis, suggesting a potential progression from substances perceived as lower risk to ones associated with higher risks. However, most individuals who began in marijuana and other low-risk subreddits did not progress to higher-risk subreddits, a finding that does not support the “causal” notions of the original gateway hypothesis. This finding aligns with the earlier-mentioned research from the National Institute on Drug Abuse in the United States, which found that while marijuana use typically precedes the use of “harder” drugs, most marijuana users do not transition to these more potent substances [6]. This consistency between web-based drug use behavior on subreddits and real-world drug consumption patterns is noteworthy. It underscores the relevance and potential utility of social media analysis as a complementary tool to traditional survey research, highlighting its capacity to offer insights into one’s behavior and progression trends. As noted by previous literature [3], there are numerous confounds that could impact the risk for progression from one substance to another, particularly escalation transitions from marijuana to higher-risk substances, given the rapid changes in accessibility, legality, and medical use of marijuana in recent decades. Furthermore, we did note instances of de-escalation transitions from higher-risk subreddits to marijuana subreddits, contrary to the gateway hypothesis and consistent with recent literature on the use of marijuana and other substances (eg, kratom) to mitigate withdrawal from higher-risk substances such as opioids [32,66,67]. Similarly, in line with this literature, we found terms related to harm reduction and recovery methods across high-risk subreddits, such as *test kit*, *get* *clean*, and *stay safe*, to be predictive of de-escalation transitions. These findings suggested that individuals might leverage a range of strategies and substances to manage and reduce their substance use, a perspective aligned with harm reduction theory.

A significant observation based on the historical behaviors was the relatively short time span within which users tend to escalate or de-escalate transitions to their risk levels. This novel finding is salient for clinicians and health care providers. By understanding these timelines, health care professionals can proactively strategize and implement interventions tailored to these predictive timelines. In addition, providing individuals with insights about the potential expectations can serve as a form of preventative care, making them aware of the potential risks and the rapidity with which transitions can occur. Our study underscored the significant potential of using machine learning and deep learning models, specifically BERT and RoBERTa, in understanding and predicting substance use transitions. The models, leveraging deep learning techniques, outperformed the traditional logistic regression model, indicating their effectiveness in capturing and learning from the complex language patterns found in substance use–related discussions. Furthermore, our findings emphasized the value of linguistic features as predictors of risk escalation and de-escalation transitions. This suggested that individuals’ narratives and discussions around their experiences, including their feelings and perceptions, could provide important clues about their trajectory of substance use. Interestingly, the prevalence of time-related terms among salient features in de-escalating transitions suggested the potential role of timing in substance use management. These findings highlight the need for timely interventions, which could potentially alter an individual’s substance use trajectory if the associated language patterns were detected early.

This study marks an initial step in understanding the role of social media data in substance use research, advocating for cautious interpretation and deeper investigation. By understanding the patterns of language use, more effective policies and therapeutic interventions could be designed to address substance use disorders. Policy makers could specifically use these findings to develop more complex and all-encompassing approaches to manage substance use disorders at both the individual and community levels, specifically taking into consideration the prevention approaches among youth as well as treatments that address polysubstance use. However, we should proceed cautiously when considering how to apply these discoveries. While our work shows that machine learning and deep learning models could be used to predict progressions of substance use, they should not be used in isolation. A thorough, multifaceted approach to addressing substance use disorders is required due to human behavior’s complexity and individual experiences’ differences. Consequently, these models should serve as one tool among many, contributing to a better understanding of substance use patterns that could inform more effective intervention strategies.

### Limitations and Future Work

We note that our study has some limitations, some of which might suggest future research. This study is limited by selection biases. We have gathered historical data only from individuals who were active on a certain number of substance-related subreddits. For example, some Reddit users might stop using web-based communities to discuss substance-related experiences or seek suggestions. This is especially true, considering the stigma and discrimination experienced by individuals with substance use disorders. The selection biases in our study are also compounded by an uneven distribution of Reddit users across different risk levels, as well as a large number of marijuana-related subreddits in risk level 2. These imbalances may reflect a nonrepresentative sample of the broader population, further skewing our results and influencing the likelihood of associations between marijuana-related discourse and escalation transitions. Thus, our observations might not be generalizable to other web-based communities or population-level trends.

It is important to note that our study adopts a broad definition of risk transitions. We cannot claim that the transitions in which a Reddit user begins posts and comments in different risk level subreddits directly correspond to clinical substance–using status. The predictors and linguistic cues that we have identified are preliminary and require further validation within a clinical setting. Our approach, while informative, cannot be directly translated into definitive assessments of substance use disorders. Future research has the potential to merge these insights with clinically validated assessments and social media data. Our method of modeling transitions might not fully capture the long-term fluctuating escalating or de-escalating behaviors of individuals. It highlights the need for more nuanced methodologies that can accurately reflect the dynamic and potentially fluctuating nature of substance use behavior over time. By doing so, a more comprehensive and generalizable understanding could be developed, providing deeper insights into substance misuse prevention, harm reduction, and recovery strategies.

In addition, our study’s approach to assigning majority labels within time bins based on the most frequent risk level presents a limitation. By focusing solely on the frequency of engagements, this method may overlook critical but less frequent high-risk interactions, potentially misrepresenting a user’s actual risk exposure. For instance, an individual’s occasional but significant high-risk activity within a predominantly low-risk engagement period could be overshadowed by the majority labeling process. Despite this limitation, it is important to underscore that our approach remains exploratory and holds significant value. It provides a foundational understanding of user trajectories in substance-related subreddit activities and offers a novel perspective on risk engagement over time. Future research might benefit from exploring methodologies that better capture the complexity of web-based behaviors, ensuring a more accurate depiction of risk levels in substance-related subreddit activities.

We highlight the limitation of the temporal restriction of our data set. The Reddit data that we used were collected from Google BigQuery spanning from 2015 to 2019. In recent years, collecting such data has become increasingly restricted due to rising privacy concerns and the evolving landscape of data protection regulations. As a consequence, researchers are often constrained to working with historical data when aiming to extract insights from such platforms. This limitation introduces the potential for outdated linguistic and behavioral references in our data set. As web-based communities and their dynamics evolve rapidly, it is possible that patterns and behaviors from 2015 to 2019 do not accurately reflect the current situation. Future studies aiming to capture a more contemporary perspective might need to explore alternative data sources that comply with current privacy standards and ethical policies.

### Conclusions

In conclusion, this study successfully used machine and deep learning models to predict future risk transitions in substance use, leveraging a rich longitudinal data set from Reddit. The fine-tuned BERT and RoBERTa models notably outperformed logistic regression models in predicting risk escalation and de-escalation transitions, with specific n-gram features playing a pivotal role in these predictions. Highlighting the critical role of language patterns in understanding substance use trajectories, the findings open avenues for future research, particularly in developing these methods with social media data to inform potential interventions in substance use behaviors.

## Appendix

Multimedia Appendix 1 Classification of subreddits by risk level with examples and definitions.

## Abbreviations

ATT: average transition time
BERT: Bidirectional encoder representations from transformers
LIWC: Linguistic Inquiry and Word Count
RoBERTa: Robustly Optimized Bidirectional Encoder Representations from Transformers Approach
RQ: research question
SAGE: Sparse Additive Generative Model
