# Supplementary material for: Examining the Gateway Hypothesis and Mapping Substance Use Pathways on Social Media: Machine Learning Approach
Source: JMIR Form Res. 2024 May 7;8:e54433. doi: 10.2196/54433 (PMC11109860; doi:10.2196/54433)
Supplement: Multimedia Appendix 1 [file formative_v8i1e54433_app1.docx]

## Multimedia Appendix 1

| Risk Level | Example Subreddit | Definition | Paraphrased posts/comments from the subreddits |
| --- | --- | --- | --- |
| 1 | *r/SMARTRecovery, r/secularsobriety, r/AtheistTwelveSteppers* | General substance use recovery or history of misuse. | In my journey with AA, my guiding force is the collective insight and consciousness of the group. Beyond the AA meetings, I draw my inspiration from humanity at large, adopting a humanistic perspective.  The knowledge shared by my peers in AA remains invaluable to me. The meetings are enriched by individuals who have maintained sobriety for many years. They offer insights into what has been successful for them, cautionary advice, and their personal narratives. This wealth of experience is something I trust and regard as a practical form of "evidence-based" guidance, given its proven effectiveness for them. [r/AtheistTwelveSteppers] |
| 2 | *r/kratom, r/cannabis, r/vaporents* | Marijuana, Kratom, and cannabinoid substances. Includes these substances due to lower risk for overdose [40], lower risk for dependence, lower risk based on traditional forms of administration (smoking, edibles), easier access and more recreational use than some other substances [41]. | In a follow-up to my previous message, I experimented with 5g of Cambodian strain this afternoon. Given my recent near-daily use of 7.2g kratom (with occasional breaks), I've noticed slight withdrawal symptoms and a mild increase in tolerance, so my expectations were modest. [...] I found myself quite energized at this lower dosage. I plan to maintain this 5g dosage, possibly considering daily or alternate-day use, which should also extend my supply. I regret not trying this approach earlier. Kratom continues to be an incredibly rewarding discovery. [r/kratom] |
| 3 | *r/pharms, r/Psychonaut, r/triponaut* | Hallucinogens/psychedelics, and misuse of prescription drugs (other than opioids [42]). Includes substances that involve significantly altered awareness or “trips” [43], the risk for acute psychiatric consequences [44] or risky behavior [45]. | So, I consumed approximately 2.5 grams of mushrooms [...] Then, after consuming just under a gram of cannabis, I went to bed. Within five minutes, I began to experience an unusual tingling sensation starting in my face and spreading throughout my body. My surroundings seemed to darken, my heart rate increased, and I felt chest pain. This episode was filled with intense fear and anxiety, leading me to believe I was experiencing a panic attack [...]. [r/Psychonaut] |
| 4 | *r/opiates,*  *r/heroin, r/fentanyl* | Opioids, benzodiazepines, stimulants. Includes substances that have a high risk for overdose and mortality [46,47], often used intravenously or other risky routes of administration, more difficult to access than other substances and more difficult to determine purity of substance [48], high potential for dependence [49]. | I'm not completely off opioids or anything; I'm still addressing my pain but not resorting to heroin to get high or flee from my difficult life, which is a significant step for me.  [r/opiates]  [...] I opted to inject heroin. The only thing I recall is getting ready to inject, and the next moment, I found myself waking up an hour later, still seated, with the needle on the floor, completely disoriented and unsure of the events that transpired. I cannot recall anything and am uncertain whether I experienced a deep state of unconsciousness or suffered an overdose the previous night.  [r/heroin] |
